# Insulin Prescribing Challenges and Physician‐Suggested Interventions: Evidence From a Tertiary Care Hospital in Lahore, Pakistan

**DOI:** 10.1002/edm2.70337

**Published:** 2026-09-11

**Authors:** Muhammad Aamir, Bazila Nafeesa, Adeel Aslam, Asma Ghulam Mustafa, Mateen Elahi, Amin Elahi, Kashif Barkat, Muhammad Umer Ashraf, Mohd Shahezwan Abd Wahab, Sumera Saeed Akhtar

**Affiliations:** ^1^ Faculty of Pharmacy University of Lahore Lahore Punjab Pakistan; ^2^ Department of Pharmacy University of Lahore Lahore Punjab Pakistan; ^3^ Department of Pharmacy Practice and Clinical Pharmacy, Faculty of Pharmacy Universiti Teknologi MARA (UiTM) Cawangan Selangor Puncak Alam Selangor Malaysia; ^4^ Department of Surgery Services Institute of Medical Sciences Lahore Punjab Pakistan; ^5^ Department of Surgery Jinnah Hospital Lahore Punjab Pakistan; ^6^ Elderly Medication and Safety Research Interest Group, Faculty of Pharmacy Universiti Teknologi MARA (UiTM) Cawangan Selangor Puncak Alam Selangor Malaysia; ^7^ Department of Primary Health Care, Faculty of Medicine University of Otago Dunedin New Zealand

**Keywords:** barriers, diabetes mellitus, feedback, insulin, insulin utilization, Pakistan, physicians

## Abstract

**Background:**

Diabetes mellitus is a major global health challenge that is controlled through insulin therapy; however, its use is limited by several barriers. This study assessed physicians' perceptions of insulin use, associated barriers and suggested improvements in a tertiary care hospital in Lahore, Pakistan.

**Methodology:**

A cross‐sectional survey was conducted at a tertiary care public sector hospital of Lahore, Pakistan, from September 2024 to February 2025. A total of 808 physicians participated. Data were collected through a questionnaire and analysed using descriptive and inferential statistics. Chi‐square, Kruskal–Wallis, Bonferroni post hoc tests and binary logistic regression were applied using IBM SPSS Version 27.

**Results:**

Among 808 physicians, 369 were males, and 439 were females. Most had 5–10 years of clinical experience, with medicine as the largest speciality. Common barriers to insulin therapy included patient resistance, hypoglycaemia, regimen complexity, cultural beliefs and limited resources. Male physicians were more likely to report barriers in both unadjusted and adjusted analyses (OR = 1.414, *p* = 0.015; AOR = 1.392, *p* = 0.024). MRCP‐qualified physicians also had higher odds of reporting barriers (OR = 1.642, *p* = 0.034; AOR = 1.657, *p* = 0.042). Physicians with 5–10 years' experience were less likely to report barriers than those with < 5 years (OR = 0.557, *p* < 0.001; AOR = 0.588, *p* < 0.001). Physicians recommended that diabetes educators and regular follow‐up can improve adherence.

**Conclusion:**

Major barriers to insulin therapy included patient resistance, inadequate education, cultural beliefs, financial constraints and fear of hypoglycaemia. Physicians emphasized the need for better patient education, regular follow‐up and improved communication to enhance insulin adherence.

## Graphical abstract

Physicians identified patient resistance, inadequate diabetes education, fear of hypoglycaemia, financial constraints, cultural beliefs and healthcare resource limitations as major barriers to insulin utilization. Improved patient education, communication, diabetes educator support and regular follow‐up were recommended to enhance insulin adherence.

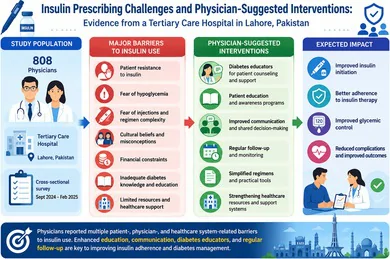

## 1 Introduction

Diabetes mellitus (DM) is a long‐term metabolic disorder characterized by persistent hyperglycaemia resulting from defects in insulin secretion, insulin action or both. It is a major global public health concern because of its increasing prevalence and its association with serious complications, including cardiovascular disease, nephropathy, neuropathy, retinopathy, disability and premature mortality [1]. DM is increasingly recognized to be a global public health burden, with the number expected to rise by another 200 million by 2040 [2]. Over half of the world's population, and about 66.6% of the total diabetic population, live in urban areas [3]. Type 2 diabetes mellitus (T2DM) is the most common form of diabetes and is strongly linked with genetic susceptibility, obesity, physical inactivity, unhealthy dietary patterns and urbanization [4]. Effective glycaemic control remains essential for reducing diabetes‐related complications and improving long‐term patient outcomes. Insulin therapy plays a central role in the management of diabetes, particularly when glycaemic targets are not achieved with lifestyle modification and oral antidiabetic agents. Insulin is essential for maintaining glucose homeostasis by promoting glucose uptake in peripheral tissues and suppressing hepatic glucose production [5].

Many of the reasons behind the inappropriate delay in the initiation of insulin therapy apply globally and are well documented. The reasons are complex, and barriers exist at the patient, physician and healthcare system level, often overlapping [6]. The factors included poor adherence to lifestyle changes (26.5%), side effects of medications (16.4%), infrequent attendance at the clinic (16.4%), poor adherence to taking medications (14.0%), lack of knowledge of diabetes (14.0%), insulin refusal (11.7%), lack of titration of tablets (7.8%) or insulin (12.5%) and social issues (10.9%) [7]. The adherence rate for oral antihyperglycaemic medication was approximately 65%–85%, and insulin adherence may be slightly lower. Healthcare system barriers contribute to 20% of therapeutic inertia, including medication availability, cost, limited resources, discontinuity of care, poor healthcare plans, high workload and time constraints and role ambiguity within the primary care team [8]. Patient‐related barriers also represent about 30% of therapeutic inertia, such as misconceptions regarding insulin risk, injection phobia, fear of weight gain, fear of hypoglycaemia, negative impact on social life and job, poor health literacy, low self‐efficacy and healthcare providers' inadequacy [9]. Primary care physicians also lacked consensus on whether patients on insulin performed self‐monitoring of blood glucose (SMBG) sufficiently to support appropriate insulin use [3]. Nearly all physicians agreed that, for most patients, education is the key to initiating insulin. However, it was noted that this education is typically provided when diabetes has progressed to the point where insulin is the only option for glucose control [10]. Despite the existence of many well‐defined targets and practice guidelines for the management of hyperglycaemia, hypertension and dyslipidaemia in patients with T2DM, clinical inertia exists due to periodic revisions of guidelines confusing healthcare providers [7]. Pakistan has 27.4 million cases of diabetes (≥ 20 years), estimated from the prevalence of diabetes at 26.3% of those living in urban areas. Therefore, to reduce this rate, proper patient education on strategies to prevent diabetes complications is an essential component of effective diabetes management and equitable care [11]. Effective diabetes management in this context requires not only access to medications but also patient education, physician counselling, culturally appropriate communication and structured follow‐up support [12, 13]. Evidence from systematic reviews suggests that behavioural intervention programmes play a crucial role in preventing and managing diabetes in adults [14]. However, initiating insulin and ensuring adherence remain challenging in clinical practice, and there is limited local evidence on the barriers physicians face when prescribing or managing insulin therapy. Understanding physicians' perspectives is important because they play a central role in initiating insulin, counselling patients, addressing misconceptions and supporting long‐term adherence. Identifying the barriers physicians face can inform practical interventions, including improved patient education, access to diabetes educators, better communication tools and health‐system support mechanisms. This study aimed to explore the barriers that physicians in Lahore, Pakistan, encounter in insulin use and to assess their feedback on strategies to improve insulin initiation, adherence and overall diabetes care.

## 2 Methodology

### 2.1 Study Design and Setting

A cross‐sectional survey‐based study was conducted. The study was conducted over 6 months, from September 2024 to February 2025, to evaluate the barriers physicians face in insulin use and their feedback. The data were gathered directly from physicians at a tertiary care public sector hospital of Lahore.

### 2.2 Study Population and Sampling

The study comprised physicians, while pharmacists, nurses and other healthcare professionals were excluded. Physicians from Internal Medicine, Endocrinology, Gastroenterology and Nephrology at a tertiary care hospital were included because they are involved in the clinical management of patients with diabetes in such settings. Eligibility was based on physicians' involvement in diabetes care rather than speciality alone. To operationalize the sampling frame, the principal investigator obtained the complete active physician roster from the hospital administration for the participating departments (Internal Medicine, Endocrinology, Gastroenterology and Nephrology). All physicians on these departmental rosters who are engaged in patient care were identified as eligible. Consequently, no additional exclusion criteria were applied beyond this operational definition, and all eligible physicians from these departments were invited to participate. The higher representation of gastroenterologists reflects physician availability during convenience sampling and does not imply that they prescribe insulin more frequently than physicians from other specialities. Physicians were recruited through direct personal contact and an online survey using Google Forms. Eligible physicians were informed about the study and invited to participate voluntarily. After obtaining informed consent, participants completed the self‐administered questionnaire in paper or electronic format, as preferred. Moreover, participants who declined to provide informed consent or submitted incomplete questionnaires were also excluded.

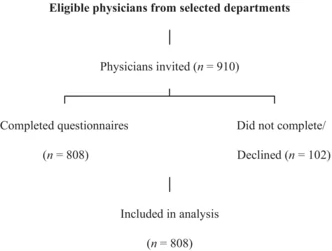

### 2.3 Sample Size Calculation

A convenience sampling technique was used to recruit eligible physicians because of feasibility and accessibility. As there was no accurate data on the number of physicians in Lahore, a sample size was calculated using the following formula by Daniels [15].

$$n=\frac{{\left(\frac{\mathrm{Za}}{2}\right)}^{2}P\;\left(1-P\right)}{d^{2}}$$

where *Z*(*a*/2) is the statistic for the 95% confidence interval (CI), which is 1.96. *P* is the prevalence, assumed to be 50%, and *d* is the precision, set at 5% (0.05).

$$n=\frac{{\left(1.96\right)}^{2}\;0.50\;\left(1-0.50\right)\;}{{\left(0.05\right)}^{2}}$$

n=384.16

The calculated minimum sample size was 384. To improve the precision of the study estimates and maximize participation among eligible physicians, 910 physicians were invited to participate. In the study, 808 completed the questionnaire, yielding an overall response rate of 88.8%. One hundred and two participants declined to provide informed consent or submitted incomplete questionnaires.

### 2.4 Study Instrument and Pilot Testing

The study's data were collected using a questionnaire developed following a thorough review of the existing literature [16, 17]. The study questionnaire consisted of three main sections designed to gather information on physicians' insights regarding barriers to insulin utilization and their feedback. All the questions were closed‐ended. The first section was demographic, comprising six closed‐ended questions that collected general information about the participants, including gender, age, primary medical speciality, years of practice and the percentage of their patients requiring insulin therapy. The second section explored various challenges and barriers that physicians encountered when prescribing insulin. It comprised 15 questions. The final section included five questions that sought physicians' opinions on areas where support was lacking, strategies to improve adherence, effective communication methods and suggestions to enhance the prescribing process and patient responses to insulin therapy. The final version of the insulin therapy questionnaire was then prepared for validity and reliability analysis. For the pilot study, a sample size of 20 subjects was recommended. A panel of 10 endocrinologists evaluated the content validity of the final version of the questionnaire. The pilot study participants (*n* = 20) were excluded from the final analysis. Data obtained during the pilot phase were used solely for questionnaire refinement and validation. To demonstrate the questionnaire's validity, content validity was assessed. The CVI for the questionnaire was 0.90. To demonstrate the questionnaire's reliability, Cronbach's alpha was calculated to assess whether the items measure a single domain. Internal consistency was assessed separately for each questionnaire domain. The Cronbach's alpha was 0.73 for the barriers section and 0.79 for the feedback section.

### 2.5 Data Analysis

After collecting the data, it was imported into Excel. Subsequently, it was coded and entered into the Statistical Package for Social Sciences (IBM SPSS) Version 27. The analysis included both descriptive and inferential statistics. Descriptive statistics, such as frequencies and percentages, summarized the data. Inferential statistics were used in the chi‐square test, with a significance level of *p* ≤ 0.05. Additionally, the Kruskal–Wallis test was applied to compare the different independent groups. For the barriers encountered by the physicians, results were categorized as 1 for those who frequently or occasionally faced the barriers and 0 for those who rarely or never faced them. Participants scoring ≥ 50% were classified as facing more barriers, while those with 50% were categorized as facing fewer barriers. The four‐point Likert responses were dichotomized into agreement and disagreement categories, following the methodology of Dolnicar et al. This approach facilitated the interpretation of the prevalence of perceived barriers and enabled comparison with previous studies [18]. For a more in‐depth analysis, binary logistic regression was applied to compute odds ratios (ORs) and adjusted odds ratios (AORs), along with their respective 95% CIs. The findings are presented in tables and figures.

### 2.6 Ethics Statement

Ethical approval for this study was obtained from the Institutional Research Ethics Committee of The University of Lahore (IREC‐2024‐30H). In addition, administrative and institutional permission for data collection from physicians was granted by a tertiary care public sector hospital of Lahore (02‐TERC/NHRC‐SZH/Ext‐SC/758). All study procedures were conducted in accordance with approved ethical guidelines and institutional requirements.

## 3 Results

### 3.1 Demographic Characteristics

Table 1 highlights key demographic insights, showing a nearly balanced gender distribution: 439 females and 369 males. Most respondents are aged 41–55 years and have 5–10 years of practice experience. Regarding education, FCPS is the most common qualification (*n* = 270), and most participants manage diabetic patients in the 11%–30% range (*n* = 308).

**TABLE 1:** Demographic characteristics of physicians.

Variable	Frequency	Per cent (%)
**Gender**		
Male	369	45.6
Female	439	54.3
**Age (years)**		
< 40	189	23.4
41–55	473	58.5
> 56	146	18.1
**Education level**		
MBBS	234	29.0
FCPS	270	33.4
MRCP	177	21.9
MRCS	127	15.7
**Practice years**		
< 5 years	228	28.2
> 10 years	144	17.8
5–10 years	436	54.0
**Percentage patients (%)**		
< 10	117	14.5
11–30	308	38.1
31–50	271	33.5
> 50	112	13.9
**Primary specialty**		
Nephrology	108	13.4
Endocrinology	197	24.4
Gastroenterology	226	28.0
Medicine	277	34.3

### 3.2 Barriers Faced by Physicians

Table 2 shows that 125 physicians with FCPS degrees occasionally faced the barrier of patients often resisting insulin therapy, whereas 111 MBBS‐qualified physicians frequently encountered this obstacle (*p* < 0.001). Similarly, 129 FCPS and 102 MBBS‐qualified physicians occasionally found it difficult to explain the differences between insulin types (e.g., short‐acting vs. long‐acting) (*p* < 0.001). Additionally, 104 FCPS‐qualified physicians occasionally encountered the barrier posed by patients' cultural beliefs to their acceptance of insulin therapy; in contrast, 89 MBBS holders experienced this barrier more often (*p* < 0.001). Furthermore, 167 FCPS holders and 108 MBBS physicians occasionally reported fear of hypoglycaemia, patient reluctance and regimen complexity as the main reasons for hesitation to initiate insulin therapy (*p* < 0.001). Lastly, 111 FCPS‐qualified physicians and 107 MRCP holders occasionally faced the barrier of insufficient resources to help patients afford insulin or manage their therapy (*p* < 0.001).

**TABLE 2:** Barriers faced by physicians with specializations.

Barriers		MBBS	FCPS	MRCP	MRCS	*p* (Kruskal–Wallis)
Patients often resist insulin therapy.	Never	8	4	1	3	**< 0.001** ***
	Rarely	40	20	11	8	
	Occasionally	75	125	91	90	
	Frequently	111	121	74	26	
Patient adherence is the primary barrier to insulin prescribing.	Never	4	5	2	4	0.450
	Rarely	37	16	17	11	
	Occasionally	101	137	86	61	
	Frequently	92	112	72	51	
Do you experience problems with patients' knowledge and understanding of insulin therapy?	Never	11	2	3	6	0.373
	Rarely	30	32	21	15	
	Occasionally	97	128	81	65	
	Frequently	96	108	72	41	
The complexity of administration is the main barrier encountered when prescribing insulin	Never	12	6	2	4	0.149
	Rarely	40	28	22	14	
	Occasionally	97	126	83	67	
	Frequently	85	110	70	42	
Is inadequate communication with patients about insulin therapy considered a barrier in clinical settings?	Never	13	6	4	6	0.519
	Rarely	36	34	23	7	
	Occasionally	80	116	84	76	
	Frequently	105	114	66	38	
Do you find that certain pharmaceutical technologies (e.g., insulin pens, pumps and continuous glucose monitors) improve insulin utilization?	Never	12	4	3	7	0.467
	Rarely	34	39	22	9	
	Occasionally	98	139	91	58	
	Frequently	90	88	61	53	
Have you encountered any issues with your patients' access to insulin formulations?	Never	10	7	4	7	0.664
	Rarely	52	38	19	11	
	Occasionally	92	156	102	78	
	Frequently	80	69	52	31	
Have you encountered difficulties conveying the differences between various types of insulin (e.g., short‐acting vs. long‐acting)?	Never	16	9	3	5	**< 0.001** ***
	Rarely	48	55	18	10	
	Occasionally	102	129	87	70	
	Frequently	68	77	69	42	
Do you think financial constraints impact a patient's ability to adhere to insulin therapy?	Never	11	4	5	6	0.350
	Rarely	51	42	24	7	
	Occasionally	87	153	96	71	
	Frequently	85	71	52	43	
Have you observed a common misconception among patients about weight gain with insulin therapy?	Never	14	6	4	5	0.248
	Rarely	47	40	21	12	
	Occasionally	94	153	98	65	
	Frequently	79	71	54	45	
Fear of needles, denial and confusion are the typical reactions of patients when you recommend insulin therapy.	Never	13	8	6	6	0.695
	Rarely	36	36	18	14	
	Occasionally	90	131	103	68	
	Frequently	95	95	50	39	
Patients' cultural beliefs also influence their acceptance of insulin therapy.	Never	16	10	6	5	**< 0.001** ***
	Rarely	50	102	69	70	
	Occasionally	79	104	62	31	
	Frequently	89	54	40	21	
Limited access, inadequate insurance, lack of education resources and delayed appointments are healthcare system barriers that can hinder effective insulin utilization among your patients.	Never	12	5	3	2	0.561
	Rarely	30	39	14	9	
	Occasionally	84	111	93	78	
	Frequently	108	115	67	38	
Fear of hypoglycaemia, patient reluctance and the complexity of regimens are the primary reasons for hesitation to initiate insulin therapy in patients.	Never	11	4	3	5	**< 0.001** ***
	Rarely	35	39	14	4	
	Occasionally	108	167	98	77	
	Frequently	80	60	62	41	
Adequate resources are available to help your patients afford insulin or manage their therapy.	Never	16	13	9	10	**< 0.001** ***
	Rarely	42	82	21	10	
	Occasionally	93	111	107	81	
	Frequently	83	64	40	26	

*Note:* Bold values indicate statistical significance.

*** *p* < 0.001.

### 3.3 Bonferroni Analysis

Bonferroni‐adjusted post hoc comparisons were performed. The Kruskal–Wallis test was employed to evaluate differences in medians across groups, and the Bonferroni adjustment was applied to control for Type I error in multiple comparisons. Regarding patients' resistance to insulin therapy, significant differences were observed between MRCS and MBBS (*p* = 0.003; Bonferroni‐adjusted *p* = 0.021) and between MRCS and MRCP (*p* = 0.001; Bonferroni‐adjusted *p* = 0.006). Additionally, a significant difference was found between MRCS and FCPS. Regarding physicians' challenges in explaining the differences between insulin types (e.g., short‐acting vs. long‐acting), notable differences were observed among MBBS, FCPS, MRCS and MRCP holders. Specifically, significant differences were observed between MBBS and MRCS (*p* < 0.001; Bonferroni‐adjusted significance at 0.002) and between FCPS and MRCP (*p* = 0.002; Bonferroni‐adjusted significance at 0.010). Other comparisons were not statistically significant. Regarding patients' cultural beliefs influencing acceptance of insulin therapy, significant differences were observed between MRCS and MBBS (*p* < 0.001; Bonferroni‐adjusted *p* < 0.001). Similarly, significant differences were observed between FCPS and MBBS (*p* < 0.001; Bonferroni‐adjusted significance at 0.001) and between MRCP and MBBS (*p* = 0.001; Bonferroni‐adjusted significance at 0.007). Regarding the barrier of fear related to hypoglycaemia, patient reluctance and complex treatment plans were the primary reasons for hesitation in initiating insulin therapy. A significant difference was found between FCPS and MRCP (*p* = 0.002; Bonferroni‐adjusted significance at 0.014), while no major differences were observed among the other groups.

Regarding the availability of resources to help patients afford insulin or manage their treatment, a significant difference was observed between FCPS and MBBS (*p* = 0.001; Bonferroni‐adjusted significance: 0.008).

### 3.4 Barriers Faced by Physicians

**FIGURE 1:**
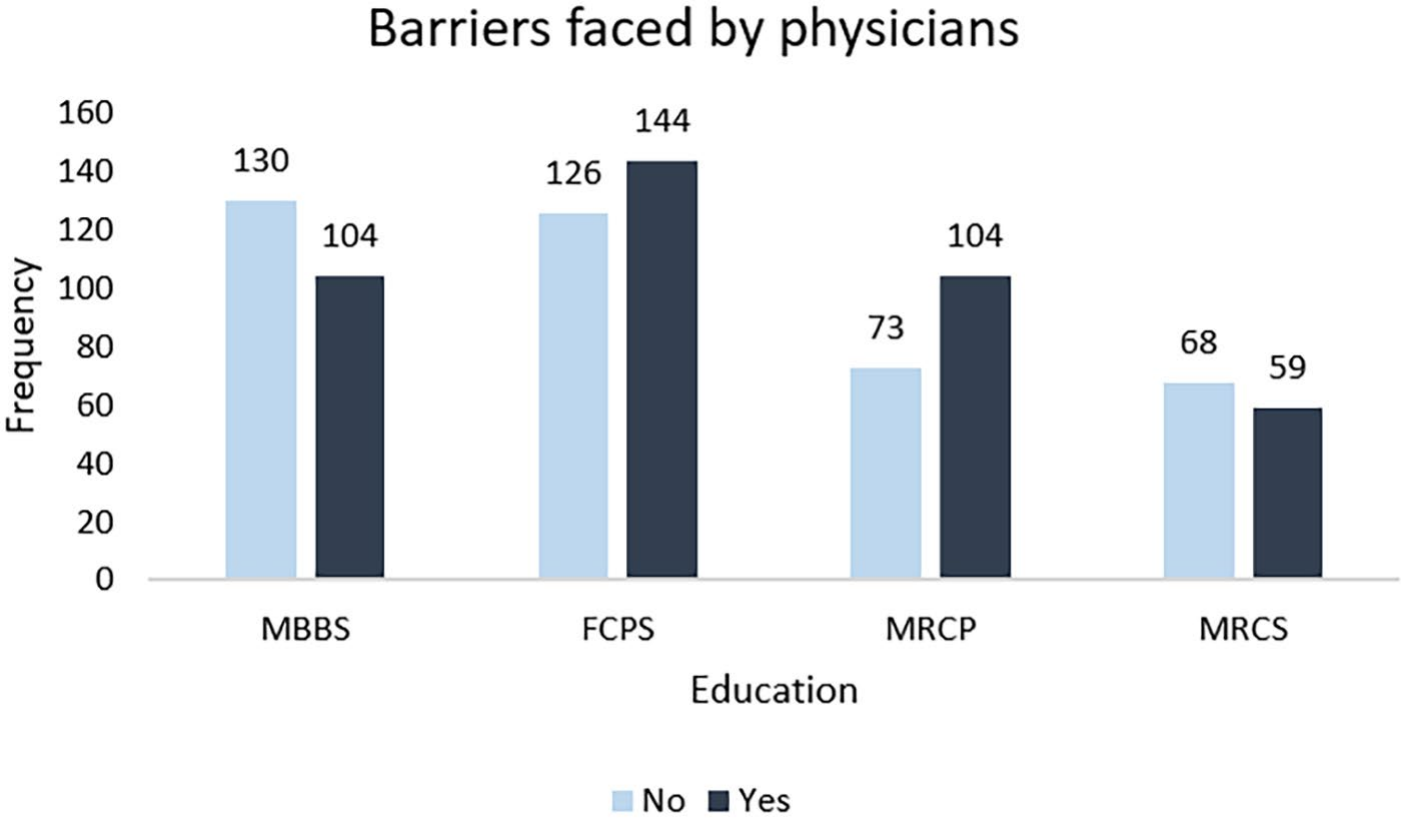
Graph showing the frequency of barriers faced by the physicians.

Figure 1 represents the barriers faced by physicians holding different degrees, including MBBS, FCPS, MRCP and MRCS. The frequency of physicians who encountered barriers and those who did not is compared within each category. Although FCPS‐certified physicians (144) accounted for the highest number of reported barriers due to their larger representation in the sample, the proportion of physicians reporting barriers was highest among MRCP‐certified physicians (*p* = 0.019).

### 3.5 Regression Analysis

Table 3 presents the regression analysis of physicians who face barriers and challenges in insulin utilization. First, across genders, males were more likely to encounter these barriers than females (OR = 1.414; *p* = 0.015, AOR = 1.392; *p* = 0.024). In terms of age, physicians aged < 40 (OR = 0.620; *p* = 0.032, AOR = 0.822; *p* = 0.506) were less likely to experience the barriers, while those holding an MRCP degree were more likely to face such barriers (OR = 1.642; *p* = 0.034, AOR = 1.657; *p* = 0.042). Although physicians in nephrology showed a higher odds ratio (OR = 1.142), this difference was not statistically significant (*p* = 0.559, AOR = 1.172; *p* = 0.503). Furthermore, physicians with 5–10 years of practice experience were significantly less likely to encounter barriers than those with < 5 years of experience.

**TABLE 3:** Regression analysis of demographics.

Demographic		Unadjusted OR (95% CI)	*p*	Adjusted OR	*p*
Gender	Male	1.414 (1.071–1.867)	**0.015** *	1.392 (1.045–1.854)	**0.024** *
	Female	Ref			
Age (years)	< 40	0.620 (0.401–0.959)	**0.032** *	0.822 (0.461–1.465)	0.506
	41–55	0.802 (0.552–1.165)	0.247	0.799 (0.501–1.274)	0.346
	> 56	Ref			
Primary specialty	Nephrology	1.142 (0.732–1.783)	0.559	1.172 (0.737–1.863)	0.503
	Endocrinology	1.012 (0.702–1.458)	0.951	0.828 (0.557–1.230)	0.349
	Gastroenterology	1.137 (0.800–1.615)	0.471	0.934 (0.636–1.372)	0.728
	Medicine	Ref			
Education	MBBS	0.922 (0.598–1.423)	0.714	1.314 (0.770–2.244)	0.317
	FCPS	1.317 (0.863–2.010)	0.202	1.612 (0.991–2.622)	0.055
	MRCP	1.642 (1.037–2.600)	**0.034** *	1.657 (1.019–2.694)	**0.042** *
	MRCS	Ref			
How many years have you been practising medicine? (years)	< 5	Ref			
	5–10	0.557 (0.403–0.771)	**< 0.001** ***	0.588 (0.397–0.871)	**0.008** **
	> 10	0.930 (0.637–1.357)	0.706	0.856 (0.533–1.375)	0.520
What percentage of patients require insulin therapy? (%)	< 10	0.745 (0.443–1.253)	0.267	0.905 (0.491–1.667)	0.748
	11–30	0.828 (0.537–1.277)	0.394	0.838 (0.499–1.410)	0.506
	31–50	1.263 (0.812–1.965)	0.301	1.284 (0.781–2.110)	0.324
	> 50	Ref			

*Note:* Bold values indicate statistical significance.

* *p* < 0.05.

** *p* < 0.01.

*** *p* < 0.001.

### 3.6 Physician Feedback

Table 4 presents feedback from physicians (MBBS, FCPS, MRCP and MRCS) regarding factors influencing understanding, adherence and prescribing practices for insulin therapy. It represents 109 FCPS and 99 MBBS degree‐qualified physicians, who suggested that patients lack access to diabetes educators in understanding insulin therapy (*p* < 0.001), followed by 117 FCPS and 99 MBBS physicians recommending regular follow‐up appointments to enhance patient adherence to insulin therapy (*p* < 0.001). Additionally, 156 FCPS and 105 MRCP‐accredited physicians reported that regular follow‐up and support are necessary to improve patient responses and adherence to insulin therapy (*p* < 0.001). Moreover, 200 FCPS holders suggested providing communication strategies to improve patient adherence, including written instructions, verbal counselling and shared decision‐making.

**TABLE 4:** Feedback from physicians on factors influencing insulin therapy.

Feedback		MBBS	FCPS	MRCP	MRCS	*p* (chi‐square test)
What type of support do you find lacking in helping patients understand insulin therapy?	Access to diabetes educators	99	109	46	18	**< 0.001** ***
	Availability of informational materials	71	69	26	20	
	Support from family members	44	43	47	27	
	Time for in‐depth discussions	20	49	58	62	
What strategy enhances patient adherence to insulin regimens?	Simplified dosing schedules	38	33	8	8	**< 0.001** ***
	Regular follow‐up appointments	99	117	41	13	
	Patient empowerment and education	69	60	49	34	
	Financial incentives	28	60	79	72	
Which communication strategy do you think improves insulin adherence?	Providing written instructions	38	14	4	4	**< 0.001** ***
	Verbal counselling	42	32	27	14	
	Shared decision‐making	41	24	31	17	
	All the above	113	200	115	92	
What improvements would you suggest to enhance the insulin prescribing process?	Streamlined electronic prescribing systems	27	14	6	2	**< 0.001** ***
	Improved patient education resources	41	25	18	11	
	Better access to insulin formulary information	37	19	23	15	
	Increased collaboration with diabetes specialists	13	13	10	8	
	All the above	116	199	120	91	
What changes would you suggest to improve patient responses and adherence to insulin therapy?	Enhanced patient education	59	26	11	8	**< 0.001** ***
	Improved access to healthcare	73	52	21	24	
	Financial support programmes	42	36	40	14	
	Regular follow‐up and support	60	156	105	81	

*Note:* Bold values indicate statistical significance.

*** *p* < 0.001.

## 4 Discussion

This study explored physicians' perceptions of barriers to insulin utilization and their recommendations for improving insulin therapy in Lahore, Pakistan. The findings indicate that physicians commonly encounter patient‐, physician‐ and health‐system‐related barriers when initiating or managing insulin therapy. The most frequently reported barriers include a lack of education (85.8%) and fear of hypoglycaemia (85.7%), influencing their insulin therapy. Other barriers consisted of complex administration (84.1%), inadequate communication (84%), fear of needles (83%), weight gain (81.5%) and financial issues (81.4%). These results align with Trinidad's research, which found that primary care physicians (PCPs) identified key patient‐perceived barriers to insulin initiation, with the most common being fear of needles (98.6%), lack of education (84.5%) and difficulty with administration (78.6%) [19]. Although fear of needles was not among the most common barriers in this study, the other barriers align with its results. A similar study among physicians in the Jazan region of Saudi Arabia reported that most of their patients fear needle injections (80.5%), and this served as a major barrier in insulin utilization [20]. In our study, the lack of education was the most common barrier physicians faced. According to another report from Saudi Arabia, most of the PCPs (68.8%) believed that the lack of education about diabetes and its complications among patients is a barrier to patients accepting insulin [20]. It shows the similarity between these two studies, in which the lack of education (85.8%) is the most common barrier faced by physicians. The DAWN study in Japan identified barriers to insulin therapy, including the risk of hypoglycaemia (*p* = 0.216), compliance with therapy (*p* = 0.179) and patients' resistance to insulin therapy (81.4%) [21]. In a study conducted in the USA among various health care workers, including physicians, nurses, pharmacists and dietitians, physicians reported a ‘lack of educational reinforcement to the patient for optimizing glycaemic control’. They only focused on glycaemic control, not on initiating insulin [22]. This result also aligned with the current study's results. An additional finding of this study was that male physicians were significantly more likely than female physicians to report barriers to insulin therapy. To our knowledge, limited evidence has specifically examined gender differences in physicians' perceptions of barriers to insulin prescribing, making direct comparison with previous studies difficult. This finding may reflect differences in clinical experiences, perceptions or practice patterns; however, the underlying reasons remain unclear. Another notable finding was that physicians with MRCP qualifications were more likely to report barriers to insulin therapy. In contrast, physicians with 5–10 years of clinical experience were less likely to report barriers than those with fewer than 5 years of experience. Together, these findings suggest that physician characteristics may influence barriers to insulin therapy. Further qualitative and longitudinal studies are needed to explore these associations and to better understand their potential implications for insulin prescribing practices and patient care. Our findings also align with a recent study conducted in Pakistan, which reported that healthcare professionals encountered multiple barriers to optimal insulin use in diabetes management. In their study, the most frequently reported barriers were time constraints (64.5%), fear of hypoglycaemia (64%), cost and limited access to insulin (63%), patient resistance (56%) and lack of training and confidence among healthcare professionals (52%). Similarly, our study identified inadequate patient education, fear of hypoglycaemia, patient misconceptions and financial constraints as major barriers to insulin therapy. However, unlike Hasan et al., whose study primarily assessed healthcare professionals' knowledge, attitudes and practices regarding insulin use, our study specifically explored physicians' perceived barriers to insulin initiation and management. It examined the demographic and professional factors associated with these perceptions in a tertiary care hospital setting [23]. In a study conducted in Turkey, the most frequently cited barrier was a lack of experience. They also devised feedback mechanisms to overcome this barrier by implementing practical applications such as simulated patients, role‐playing, monitoring experienced physicians' patient interviews and consulting patients with an expert, which can be organized within continuous professional education programmes. However, fear of injections ranked second in importance among the barriers in their study. The physicians of the current study also faced this barrier [24]. Our findings show that fear of hypoglycaemia (85.7%) is a frequently reported barrier among physicians; a similar study from Nigeria found that fear of hypoglycaemia (81.3%) was the most frequently reported barrier to insulin initiation among respondents. The second most common barrier faced by their physicians was refusal of patients (79.7%), along with fear of needles (65.6%) and fear of weight gain (23.3%). These results also align with the current study. Still, the most common barrier faced by physicians was a lack of education, which is uncommon in Nigeria. This may be due to Pakistan being a developing country and having literacy rates lower than those of these countries [25]. According to another study conducted in Malaysia, healthcare professionals found that patients' fear of side effects, such as hypoglycaemia and weight gain, was a common barrier faced by patients. This barrier was also a major one in the current study. Another major barrier faced by the physicians was misconceptions of severe complications after insulin therapy [26]. In the current study, physicians (85.8%) frequently faced the barrier of insufficient educational resources to help patients manage their therapy. A subsequent study in the Caribbean Island of Barbados reported that most doctors (68%) felt that patient education on insulin use was uncomplicated, and 36% did not consider insulin initiation among the most difficult aspects of managing patients with Type 2 diabetes [27]. Lack of education or low educational levels is one of the major barriers physicians face. Another study conducted among Asian patients in Singapore stated that educational level was significantly associated with willingness to use insulin therapy. Our findings agree with those of Al Alidrisi et al., who reported that physicians perceived poor adherence, lack of patient motivation, fear of hypoglycaemia, socio‐economic challenges and inadequate follow‐up as major barriers to insulin initiation. Both studies emphasize the importance of addressing patient‐related concerns and improving healthcare support. However, while Al Alidrisi et al. focused on barriers to insulin initiation among doctors and patients, our study provides broader insights into insulin utilization, including prescribing challenges and physicians' recommendations to improve insulin use [28]. This highlights the correspondence of the current study with previous findings. Consistent with the present findings, Galdón Sanz‐Pastor et al. reported that healthcare professionals face several challenges when initiating insulin therapy, including concerns about hypoglycaemia, treatment complexity, inadequate patient education and therapeutic inertia. Similarly, physicians in the current study identified fear of hypoglycaemia, lack of educational resources, inadequate communication and complexity of insulin administration as major barriers. The overlap in findings suggests that both physician‐related and healthcare system‐related factors continue to hinder timely insulin initiation in patients with Type 2 diabetes [29]. Consistent with the current study, Newson et al. found that healthcare providers considered patient non‐adherence, forgetfulness and lifestyle‐related challenges as major contributors to suboptimal insulin use. Their findings also underscore the importance of individualized education and clinical support in improving insulin management. However, unlike their study, which included healthcare providers managing both Type 1 and Type 2 diabetes, our study specifically explored physicians' perspectives on insulin utilization barriers and improvement strategies [30]. According to another study in Singapore, fear of needles, diabetes education and hypoglycaemia are among the major barriers identified (26), which align with the primary barriers highlighted in the current study. Not enough staff and limited time for patient education are common reasons for delays in starting insulin and for patients not learning enough about diabetes [31]. These factors contribute to a lack of patient education. Our findings highlight barriers faced by physicians, patients and the broader healthcare system. Most of these common barriers among all the studies were fear of needles, lack of education, fear of hypoglycaemia and a few others. Addressing these challenges through targeted interventions and appropriate strategies is clinically significant. These strategies involve providing adequate physician training and enhancing patient education to support effective diabetes management. To improve this, Pakistan's telecommunications industry is undergoing rapid development, enhancing connectivity across the country. Furthermore, a systematic review by Aslam et al. (2025) [14] provides evidence supporting the effectiveness of behavioural intervention programmes, including patient education, counselling and structured follow‐up, in preventing and managing diabetes in adult populations. Also, screening initiatives like the Risk Assessment of Pakistani Individuals for Diabetes (RAPID) aim to identify high‐risk individuals early [32]. Such efforts can lead to better glycaemic control and a consequent reduction in the burden of diabetes‐related complications.

### 4.1 Limitations

This study also has some limitations. The use of a convenience sampling technique may limit the generalizability of the findings. Data were self‐reported, which may introduce recall or social desirability bias. The inclusion of an ‘All of the above’ response category may have introduced overlap with individual response options, which should be considered when interpreting the associated chi‐square analyses. Also, the regression analysis did not include a formal assessment of multicollinearity, model fit indices or potential clustering effects by department or speciality, which may affect the robustness of the reported associations. As data were collected only from physicians, the identified barriers reflect physicians' perceptions and may not fully represent patients' actual experiences or barriers to insulin therapy. Additionally, dichotomizing the four‐point Likert scale may have reduced the variability and detail of participants' responses, which should be considered when interpreting the findings. The cross‐sectional design allows identification of associations but does not establish causality. In addition, the study was conducted in Lahore, and the findings may not fully represent physicians working in other regions of Pakistan or in rural healthcare settings.

## 5 Conclusion

This study identifies a range of physician‐perceived barriers to insulin initiation and management in a tertiary care setting in Lahore, Pakistan, including patient resistance, financial constraints, cultural beliefs, fear of needles, hypoglycaemia and inadequate patient education resources. The likelihood of encountering these barriers was significantly associated with physician gender, qualification and years of clinical experience. Physicians with < 5 years of experience reported the most barriers, while male physicians and those with MRCP qualifications were more likely to report barriers to insulin therapy, highlighting the need for targeted professional development and further investigation into factors influencing physicians' perceptions across different demographic and professional groups. Physicians recommended regular follow‐up, improved access to diabetes educators and enhanced patient communication strategies as priority interventions. Future studies should evaluate the effectiveness of educational and organizational interventions in reducing the barriers identified in the present study to improve insulin utilization and reduce the burden of diabetes‐related complications in Pakistan. While the identified strategies were commonly recommended by participants, their effectiveness was not evaluated in the present study and should be investigated in future intervention‐based research.

## Author Contributions

**Muhammad Aamir:** conceptualization, methodology, formal analysis, writing – review and editing, writing – original draft. **Bazila Nafeesa:** investigation, data curation, writing – original draft, visualization. **Adeel Aslam:** methodology, validation, writing – review and editing, supervision. **Asma Ghulam Mustafa:** investigation, resources, writing – review and editing, writing – original draft. **Mateen Elahi:** resources, writing – review and editing, project administration. **Amin Elahi:** formal analysis, software, data curation. **Kashif Barkat:** visualization, validation, writing – review and editing. **Muhammad Umer Ashraf:** methodology, formal analysis, writing – original draft. **Mohd Shahezwan Abd Wahab:** conceptualization, writing – review and editing, validation, supervision. **Sumera Saeed Akhtar:** conceptualization, methodology, formal analysis, project administration, supervision, writing – review and editing.

## Funding

The authors have nothing to report.

## Conflicts of Interest

The authors declare no conflicts of interest.

## Data Availability

The data that support the findings of this study are available from the corresponding author upon reasonable request.
